# A Self-Assembled G-Quadruplex/Hemin DNAzyme-Driven DNA Walker Strategy for Sensitive and Rapid Detection of Lead Ions Based on Rolling Circle Amplification

**DOI:** 10.3390/bios13080761

**Published:** 2023-07-26

**Authors:** Yuhan Wang, Jiaxuan Xiao, Xiaona Lin, Amira Waheed, Ayyanu Ravikumar, Zhen Zhang, Yanmin Zou, Chengshui Chen

**Affiliations:** 1 School of Emergency Management, School of the Environment and Safety Engineering, Jiangsu University, Zhenjiang 212013, China; 2 Department of Pulmonary and Critical Care Medicine, The Quzhou Affiliated Hospital of Wenzhou Medical University, Quzhou People’s Hospital, Quzhou 324000, China; 3 Key Laboratory of Interventional Pulmonology of Zhejiang Province, Department of Pulmonary and Critical Care Medicine, The First Affiliated Hospital of Wenzhou Medical University, Wenzhou 325000, China; 4 School of Pharmacy, Jiangsu University, Zhenjiang 212013, China

**Keywords:** biosensor, rapid detection, signal amplification, rolling circle amplification, G-quadruplex

## Abstract

Herein, a sensitive biosensor is constructed based on a novel rolling circle amplification (RCA) for colorimetric quantification of lead ion (Pb^2+^). At the detection system, GR5 DNAzymes are modified on the surface of an immunomagnetic bead, and Pb^2+^ is captured by the aptamer, inducing the disintegration of the GR5 DNAzyme and the release of the DNA walker. After the introduction of the template DNA, T4 DNA ligase, and phi29 DNA polymerase, an RCA is initiated for the sensitivity improvement of this method. Moreover, a G4-hemin DNAzyme is formed as a colorimetric signal, owing to its peroxide-like activity to catalyze the TMB-H_2_O_2_ substrate. Under the optimized conditions, the limit of detection (LOD) of this fabricated biosensor could reach 3.3 pM for Pb^2+^ with a concentration in the range of 0.01–1000 nM. Furthermore, the results of real samples analysis demonstrate its satisfactory accuracy, implying its great potential in the rapid detection of heavy metals in the environment.

## 1. Introduction

Lead (Pb), a generic class of heavy metal, has been widely used in the manufacture of solder, batteries, and radiation protection equipment [1,2,3] due to its unique advantages, including low melting point, corrosion resistance, and strong radiation absorption. However, extensive use has brought about widespread environmental occurrences and influences through a wide range of processes and pathways [4,5]. Lead has been found in various environmental sources, such as surface water [6], groundwater [7], soil sediments [8], and aquatic organisms [9]. Even a very small amount of lead in humans can cause irreversible damage to human health [10,11,12]. One major problem is that exposure to Pb can modify sex hormones, resulting in chromatin destabilization, congenital disabilities, and atrophy of the seminiferous tubule [13,14,15]. Therefore, it is imperative to develop a proficient approach to quickly detect Pb^2+^ in environmental samples to facilitate risk assessments [10].

In recent years, a multitude of analytical techniques have emerged for the identification and quantification of Pb^2+^, including UV-spectrophotometry [16], inductively coupled plasma mass spectrometry (ICP-MS) [17], atomic fluorescence spectroscopy (AFS) [18], and atomic absorption spectroscopy (AAS) [19]. However, these analytical methods possess certain limitations, such as laborious sample pretreatment procedures, the necessity of substantial quantities of organic solvents for enrichment, and the reliance on costly instrumentation. Therefore, colorimetric sensors have shown great promise as a more effective method [20,21] and DNA-based sensors are expected to become important for the detection of Pb^2+^ [22,23,24].

Heavy-metal-dependent DNAzymes are significantly used for metal ions detection [22,23,25,26,27] due to their high catalytic activity and metal ion selectivity. Hence, a DNAzyme-based sensor has great potential for Pb^2+^ detection [28,29]. However, realizing ultrasensitive detection with DNAzymes alone is still difficult. In the past few years, a diverse range of nucleic acid amplification strategies [30,31] have been employed in sensing architectures to improve the concentration of the target nucleic acids, such as polymerase chain reaction (PCR) [32], strand displacement amplification (SDA) [33], hybridization chain reaction (HCR) [34], and rolling circle amplification (RCA) [35]. Among these methods, RCA represents a potent and efficient isothermal enzymatic amplification strategy [36]. The gentle reaction conditions, stability, and efficiency in complex environments render it a simple and reliable nucleic acid amplification method [37]. RCA reaction involves a circular template, leading to the generation of numbers of reduplicated sequences, synthesizing ultralong single-strand DNA, and improving remarkably the sensitivity of the analytical method. Pang et al. have successfully developed a highly sensitive electrochemical biosensor for detecting Pb^2+^. This biosensor utilizes a combination of RCA and DNA-AgNCs amplification strategies. The biosensor demonstrated an impressively low detection limit of 0.3 pM, along with a wide linear range spanning from 1 pM to 10 μM [38]. On the other hand, Yuan et al. have proposed an innovative and ultrasensitive detection method for Pb^2+^. Their method utilizes a fluorescent assay and relies on an RCA-assisted multisite-SDR signal amplification strategy [39]. The strategy exhibited excellent performance, exhibiting a linear range from 0.1 to 50 nM and achieving a detection limit as low as 0.03 nM. Furthermore, Guo et al. developed an electrochemical sensing system for Pb^2+^ by utilizing AI-AuNPs modification and improved RCA [40]. This sensing strategy demonstrated a detection range spanning from 1 pM to 1 μM and achieved a detection limit of 290 fM. RCA and DNA walkers [41] are both signal amplification strategies of significant importance. The DNA walkers, recognized as signal amplifiers, can be integrated with diverse amplification methodologies to enhance signal transduction and amplify biosensor sensing signals, enabling dual signal amplification [41,42].

This study developed a colorimetric method for the ultrasensitive detection of Pb^2+^ by employing a Pb^2+^-specific DNAzyme and G4/hemin produced by RCA. The DNA walker played a role in target recognition and signal amplification. The DNAzyme was activated by Pb^2+^, cleaving the substrate strand (SS) into two fragments at its “rA” site, releasing the cDNA as a primer for subsequent RCA. The cDNA can hybridize with the template strand (TEMP) to form a circular DNA (circ-DNA). Under the presence of phi29 DNA polymerase and dNTPs, the circ-DNA underwent an RCA reaction and elongated the cDNA. The resulting RCA products contain many G-quadruplex (G4) structures that can bind hemin to form G4/hemin, catalyzing the colorless TMB-H_2_O_2_ substrate to turn blue and producing an enhanced colorimetric signal. A novel approach is employed in the development of this sensor, leveraging the Pb^2+^-specific DNAzyme and the G4/hemin generated through RCA to enable the detection of Pb^2+^. Ultimately, this method was successfully applied for the detection of Pb^2+^ in real samples.

## 2. Materials and Methods

### 2.1. Chemicals

The DNA samples used in this study were obtained from Sangon Biotech (Shanghai, China) for experimental purposes; the DNA sequences and modifications are listed in Table 1. All DNA samples were purified by HPLC, stocked in 1× TE buffer (pH = 7.8), and stored at −20 °C.

T4 DNA ligase and phi29 DNA polymerase were purchased from Beyotime Biotech (Shanghai, China). Beyotime Biotech also provided a 10× T4 DNA ligation buffer and 10× phi29 DNA polymerase reaction buffer. Streptavidin immunomagnetic beads (IMBs, 10 mg/mL, 0.5 μm), dNTPs, and BSA were purchased from Sangon Biotech (Shanghai, China). Hemin, 3, 3′, 5, 5′-tetramethylbenzidine (TMB), and hydrogen peroxide (H_2_O_2_) were purchased from Beyotime Biotech. All other chemical reagents were bought from Sangon Biotech and were of analytical grade. All solutions in this study were prepared using ultrapure water sourced from Milli-Q (18 MΩ, Merck Millipore, Shanghai, China). The buffers used in this method are listed as follows:

Binding Buffer (10×): 100 mM Tris-HCl, 500 mM MgCl_2_, 500 mM NaCl.

Enzyme Activity Buffer: 100 mM Tris Base, 120 mM NaCl, 10 mM MgCl_2_, 100 mM KCl.

Substrate Buffer: 200 mM Na_2_HPO_4_, 100 mM citric acid.

### 2.2. Preparation of DNA Walker-IMBs

A volume of 2 μL of 10 μM biotin-modified SS and 2 μL of 1 μM enzyme strand (ES) were combined with 1× binding buffer to obtain a total volume of 20 μL. The mixture was heated at 95 °C for 5 min, followed by cooling to room temperature, resulting in the formation of the GR5 DNAzyme duplex. The 2 μL suspension of IMBs underwent three washes with 1× PBS. Next, a 20 μL solution of the GR5 DNAzyme duplex was added to the cleaned IMBs and gently shaken for 1 h, resulting in the formation of DNA walker-IMBs. The DNA walker-IMBs were magnetically separated and subsequently washed three times with 1× PBS to eliminate any unbound DNA. Finally, the DNA walker-IMBs were resuspended in 5 μL of 1× PBS and stored at 4 °C until subsequent experiments.

### 2.3. RCA Reaction Signal Amplification and Pb^2+^ Detection

First, 10 μL of Pb^2+^ at different concentrations were introduced into the 5 μL DNA walker-IMBs system, followed by incubation at 37 °C with gentle shaking for 1 h. After magnetic separation, the supernatant containing the released cDNA was collected. Next, 1 μL of the template (100 μM), 5 μL of 800 mM NaCl, and 4 μL of H_2_O were added to 10 μL of the supernatant. The mixed solution was heated at 95 °C for 5 min and then slowly cooled to room temperature to facilitate the hybridization of the TEMP and cDNA, resulting in the cyclization of TEMP and the formation of the circ-DNA precursor.

For the ligation reaction, a 50 μL mixture was prepared, containing 20 μL of the circ-DNA precursor, 5 μL of 10× T4 DNA ligation buffer, and 24.8 μL H_2_O. After mixing the above ingredients, 0.2 μL of 1000 U/μL T4 DNA ligase was added. The mixture was incubated at room temperature for 40 min, followed by inactivation of the T4 DNA ligase through heating at 70 °C for 10 min. To initiate the RCA reaction, 10 μL of 10× phi29 buffer, 1 μL of 10 U/μL phi29 DNA polymerase, 10 μL of 800 mM NaCl, 1 μL of 40 mg/mL BSA, and 2 μL of 10 mM dNTPs were added to the reaction mixture to reach a final volume of 100 μL. The mixture was incubated at 30 °C for 1 h to allow for rolling circle amplification. The resulting rolling circle amplification product (RCP) was then inactivated by heating at 70 °C for 10 min.

For the preparation of RCP G4/hemin, a simple self-assembly strategy was employed. Specifically, 20 μL of RCP was mixed with 20 μL of 120 μM hemin and 40 μL of enzyme activity buffer. The mixture was heated at 95 °C for 5 min and then incubated at 37 °C for 1 h. Subsequently, 160 μL of the enzyme-substrate solution (1 mL substrate buffer containing 100 μL of 10 mM TMB and 10 μL of 0.75% H_2_O_2_) was mixed with 80 μL of RCP and incubated at 37 °C for 5 min. Finally, the absorbance signals were measured at 650 nm using a multimode microplate reader.

## 3. Results and Discussion

### 3.1. Detection Strategy of the Biosensor

**Scheme 1:**
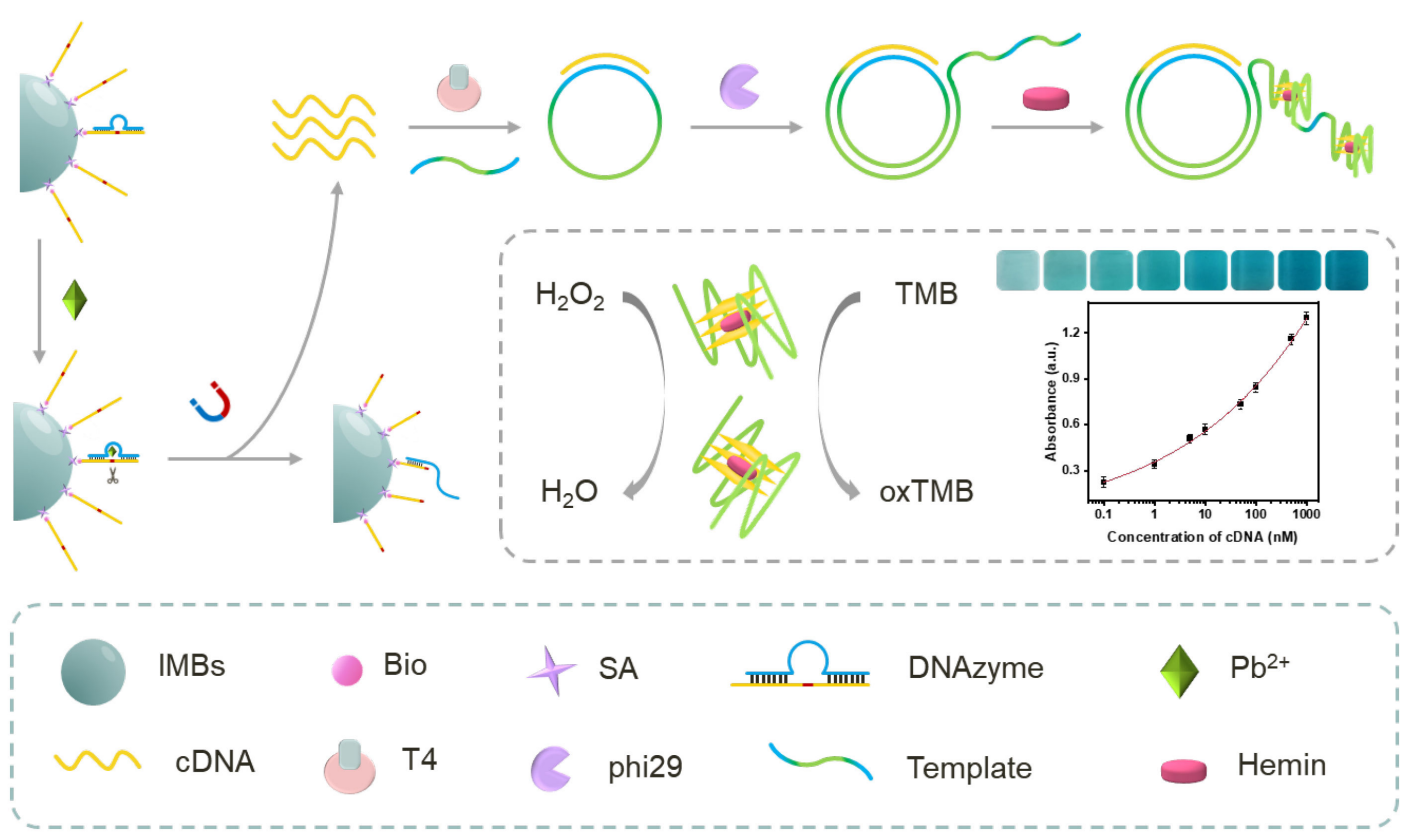
Schematic illustration of the Pb^2+^ colorimetric biosensor.

Herein, a sensitive colorimetric biosensor was proposed for Pb^2+^ detection based on RCA amplification to form repeated G4/hemin as a signal molecule (Scheme 1). To detect the Pb^2+^, GR5 DNAzyme was chosen as the DNAzyme for Pb^2+^ recognition, owing to its specific identification with Pb^2+^ compared to other Pb^2+^-based DNAzymes (8-17 DNAzyme) [43,44,45]. In the presence of Pb^2+^, the cleavage activity of GR5 DNAzyme was activated, leading to the stem–loop structure destruction and releasing segment DNA (cDNA). After that, ES can bind with other SS, initiating a new round of DNAzyme cleavage and generating additional cDNA. Then, magnetic separation was performed to collect the supernatant containing the released cDNA, which is complementary with the template DNA (TEMP) to form the circular DNA (circ-DNA) precursor. Under the catalytic action of T4 DNA ligase on its substrate TEMP, the two ends of TEMP were ligated, thereby forming a circ-DNA. Subsequently, the cDNA served as a primer to initiate the RCA with the addition of phi29 DNA polymerase and dNTPs, resulting in the generation of a long single-strand DNA (RCP) containing a repetitive G-quadruplexes (G4) sequence. In the presence of K^+^, the RCP can bind with hemin to fold into G4/hemin DNAzymes, catalyzing the TMB/H_2_O_2_ to generate a colorimetric signal.

### 3.2. Feasibility of the DNAzyme and DNA Walker

**Figure 1:**
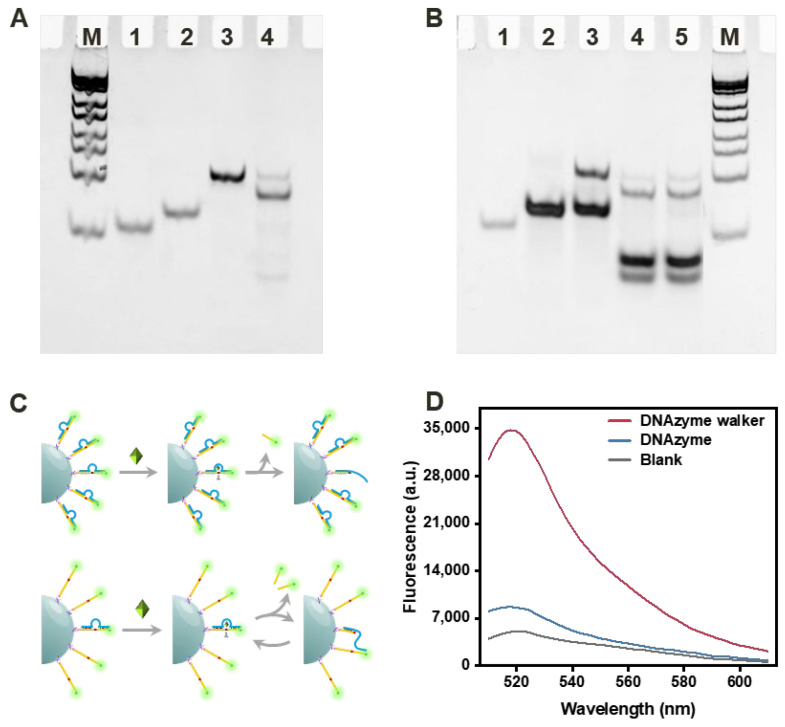
Feasibility of the GR5 DNAzyme and DNA walker. (**A**) EMSA of GR5 DNAzyme (SS:ES = 1:1) assembly and cleavage in 2 μM Pb^2+^. M: DNA marker; Lane 1: SS; Lane 2: ES; Lane 3: SS + ES; Lane 4: SS + ES + Pb^2+^. (**B**) EMSA of DNA walker (SS:ES = 10:1) with different concentrations of Pb^2+^ (0, 5, 20 μM). Lane 1: ES; Lane 2: SS; Lane 3: SS + ES; Lane 4: SS + ES + 5 μM Pb^2+^; Lane 5: SS + ES + 20 μM Pb^2+^. (**C**) Fluorescence assays scheme and (**D**) Fluorescence intensity results of IMBs-DNAzyme (SS:ES = 1:1) and IMBs-DNA walker (SS:ES = 10:1) with 10 μM Pb^2+^.

In order to investigate the assembly and cleavage of the DNAzyme and DNA walker, an electrophoretic mobility shift assay (EMSA) and fluorescence assays were employed as analytical techniques. The 8% polyacrylamide gel electrophoresis (PAGE) image of DNAzyme is shown in Figure 1A; line 3 displays a band that migrated slower than the bands of the substrate strand (SS, line 1) and the enzyme strand (ES, line 2), indicating the successful assembly of the DNAzyme duplex. In the presence of Pb^2+^, two bands (line 4) appeared, representing the DNAzyme subunit and the released cDNA, confirming the cleavage of the DNAzyme and the release of cDNA.

To evaluate the functionality of the DNA walker, DNAzymes were synthesized using a 10:1 molar ratio of SS and ES, and various concentrations of Pb^2+^ were added in the absence of immobilized IMBs. In the 8% PAGE image shown in Figure 1B, line 3 shows two bands due to the excess SS, and upon the addition of Pb^2+^, four bands appeared in line 4 and line 5, corresponding to excess DNAzymes, DNAzyme subunits, SS fragments, and cDNA. The presence of SS fragments and cDNA brands, along with the reduction or even disappearance of SS, indicate the feasibility of the DNA walker.

Furthermore, fluorescence assays were conducted using FAM-labeled SS to further evaluate the performance of the IMBs-DNA walker system. Firstly, FAM-labeled SS and ES were modified on the surface of the IMBs in ratios of 1:1 and 10:1, respectively, and the concentration of SS was ensured to be the same. Then 10 μM Pb^2+^ was added to verify the feasibility and signal amplification efficiency of the IMBs-DNA walker system. The fluorescence assay scheme and fluorescence intensity results are displayed in Figure 1C,D; compared to the regular DNAzyme, the DNA walker exhibited a significant fluorescence enhancement upon the introduction of Pb^2+^, which could be distinctly discriminated from the blank control. This indicates that the IMBs-DNA walker successfully cleaved and released cDNA. Additionally, Figure S1 demonstrates that, as the concentration of Pb^2+^ increased, the fluorescence intensity gradually enhanced, indicating that the IMBs-DNA walker can generate distinct intensity responses at different concentrations.

### 3.3. Feasibility of the RCA Reaction and Verification of G4/Hemin

**Figure 2:**
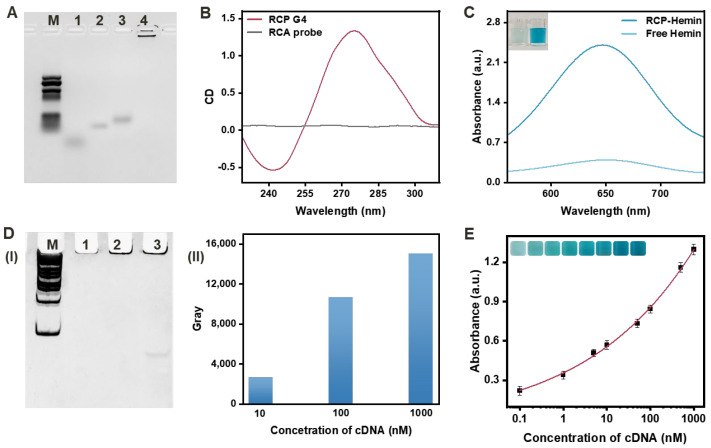
(**A**) EMSA for the RCA development. M: DNA marker; Lane 1: cDNA; Lane 2: TEMP; Lane 3: circ-DNA; Lane 4: RCP. (**B**) CD spectroscopy for the G-quadruplex. (**C**) UV- absorption spectra and the corresponding optical photographs (the inset) of the TMB-H_2_O_2_ substrate system with RCP-hemin and free hemin. (**D**) The RCP with different concentrations of cDNA (10, 100, 1000 nM): (**I**) EMSA for the RCP. M: DNA marker; Lane 1: RCP with 10 nM cDNA; Lane 2: RCP with 100 nM cDNA; Lane 3: RCP with 1000 nM cDNA. (**II**) The gray value of the RCP bands. (**E**) UV-absorption spectra and the corresponding optional photographs (the inset) of the TMB-H_2_O_2_ substrate system with RCP produced by amplification of cDNA at different concentrations (0.1, 1, 5, 10, 50, 100, 500, 1000 nM). The error bars represent the standard deviation of three measurements.

The RCA reaction and RCP were verified through EMSA and circular dichroism spectroscopy (CD). The 2% agarose gel electrophoresis (AGE) results are shown in Figure 2A, wherein the circ-DNA band (line 3) exhibited slower migration compared to the bands of cDNA (line 1) and TEMP (line 2). When the circ-DNA reacted with phi29 DNA polymerase and dNTPs (line 4), a bright band was observed at the sample pore due to the inhibition of movement caused by the increased length of the RCP single-strand DNA. These results demonstrated that the cDNA could trigger the RCA reaction, leading to the amplification of a long single-strand DNA.

The CD analysis results of RCP, as shown in Figure 2B, revealed a negative peak near 240 nm and a positive peak near 270 nm in the presence of circ-DNA, phi29 DNA polymerase, and dNTPs. However, no significant peak was observed when only the RCA probe was present, indicating the successful generation of the G-quadruplex structure through RCA.

To verify the peroxidase-like catalytic capacity of the RCP G4-hemin, the TMB-H_2_O_2_ substrate was employed as the model. The resulting absorbance spectra are depicted in Figure 2C. Both free hemin and the RCP G4-hemin structure catalyzed the oxidation of TMB, producing a blue product (ox TMB) with absorbance at 650 nm. The RCP G4-hemin structure exhibited an obvious colorimetric signal, which could be clearly distinguished from free hemin, indicating that the G4 structure of the RCP enhanced the catalytic effect of hemin.

The amplification results of varying cDNA concentrations are presented in Figure 2(DI,II). With increasing cDNA concentration, the bands in the gel gradually intensified in color, and the gray value also increased. The color of the reaction solution gradually shifted from light blue to dark blue, enabling easy visual identification even with the naked eye (Figure 2E). These results confirm that the RCA reaction triggered by cDNA could effectively respond to different concentrations.

### 3.4. Optimization of Detection Conditions

**Figure 3:**
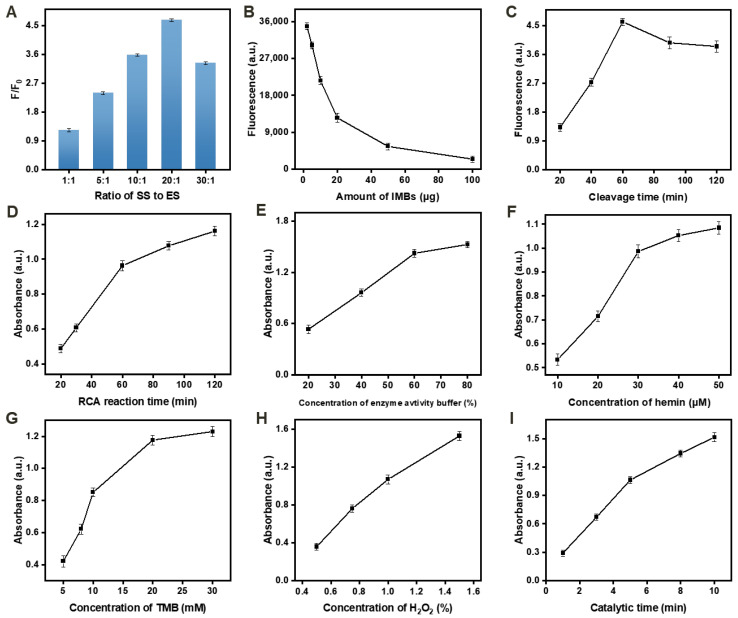
Pb^2+^ detection assay parameter optimization. (**A**) Effect of the ratio of SS to ES. (**B**) Effect of the concentration of IMBs. (**C**) Effect of the DNA walker cleavage time. (**D**) Effect of the RCA reaction time. (**E**) Effect of the concentration of enzyme activity buffer. (**F**) Effect of the concentration of hemin. (**G**) Effect of the concentration of TMB. (**H**) Effect of the concentration of H_2_O_2_. (**I**) Effect of the catalytic time. The concentration of cDNA used in these tests was 50 nM. All the tests were repeated in triplicate.

To optimize the detection performance, several crucial parameters were analyzed, including the mole ratio of SS to ES, the maximum loading capacity of SS on IMBs, DNAzyme walker digestion time, RCA reaction time, the concentration of enzyme activity buffer, hemin, TMB, and H_2_O_2_, and the colorimetric catalytic time. The effects of these parameters were evaluated as follows:

Figure 3A illustrates the effects of changing the mole ratio of SS to ES. The fluorescence intensity reached a plateau when the mole ratio was 20:1. This was due to the low mole ratio, resulting in low cleavage efficiency of DNAzyme, whereas a higher mole ratio led to the steric hindrance and reduced the cleavage efficiency of the DNAzyme on the surface of IMBs.

Figure 3B displays the effect of varying the loading capacity of IMBs with 20 μL of FAM-SS (1 μM). The highest fluorescence intensity was observed when the IMB amount was 2 μg, indicating optimal loading capacity.

Figure 3C,D show the optimized DNA walker cleavage time and RCA reaction time, which were found to be 60 min for both processes. These times yielded the most efficient cleavage and amplification.

The effects of the concentration of the enzyme activity buffer, hemin, TMB, and H_2_O_2_ were evaluated, as depicted in Figure 3E–H. The best absorbance was obtained when the concentrations of enzyme activity buffer, hemin, TMB, and H_2_O_2_ were at 60%, 30 μM, 20 mM, and 1%, respectively. Excessive substrate concentrations may result in excessively dark color and rapid peroxidation of ox TMB.

Finally, the catalytic reaction time of TMB/H_2_O_2_ was achieved at 5 min (Figure 3I) to achieve the most prominent catalytic effect in this system.

### 3.5. Sensitivity, Selectivity, and Reproducibility of the Biosensor for Pb^2+^ Detection

**Figure 4:**
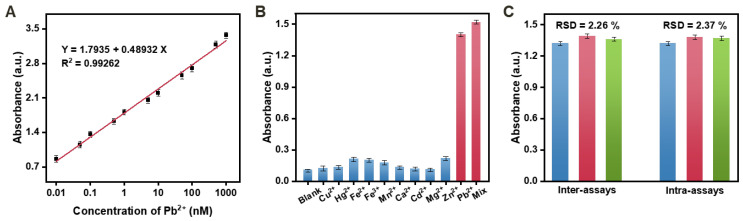
(**A**) Sensitivity of the proposed biosensor for Pb^2+^. (**B**) Selectivity of the proposed biosensor for Pb^2+^ (0.1 nM) against other interference ions (each at 10 nM). (**C**) Reproducibility of the biosensor in three parallel inter-assays and intra-assays. Error bars: SD, *n* = 3.

Under optimal conditions, the performance of the method was evaluated by introducing different concentrations of the target Pb^2+^. The results demonstrated a gradual increase in absorbance as the Pb^2+^ concentration ranged from 0.01 to 1000 nM, as depicted in Figure 4A. The calibration equation was determined as Y = 1.7935 + 0.48932X (R^2^ = 0.99262), indicating the reliability of the method for quantifying Pb^2+^ concentrations. The detection limit was determined to be 3.3 pM based on 3 σ/slope (σ = standard deviation of the blank samples). Comparative analysis with previous Pb^2+^ detection methods (Table 2) revealed that this method exhibited higher amplification efficiency and enhanced sensitivity. These findings underscore the improved performance and potential utility of the proposed method for sensitive and accurate Pb^2+^ detection.

The exploration of selectivity was a crucial aspect of the proposed biosensor. To assess selectivity, various metal ions, including Cu^2+^, Hg^2+^, Fe^2+^, Fe^3+^, Mn^2+^, Ca^2+^, Cd^2+^, Mg^2+^, and Zn^2+^, and a mixture of these ions, were tested under identical conditions for comparison. As shown in Figure 4B, only the Pb^2+^ sample (0.1 nM) exhibited a significant signal response. In contrast, the absorbance signals generated by the other non-target metal ions, even at concentrations 10 times higher than that of Pb^2+^, were comparable to the bank sample. These findings indicate the high selectivity and anti-interference capability of the developed biosensor for Pb^2+^ detection. Moreover, there was no discernible difference in the absorbance between the mixture sample containing Pb^2+^, Cu^2+^, Hg^2+^, Fe^2+^, Fe^3+^, Mn^2+^, Ca^2+^, Cd^2+^, Mg^2+^, and Zn^2+^ with Pb^2+^ alone. This observation further confirms the high specificity of the fabricated biosensor for Pb^2+^ detection, which can be primarily attributed to the strong affinity between the DNAzyme and Pb^2+^.

To assess the reproducibility of the developed biosensor, multiple measurements were performed to evaluate the consistency and reliability of the results. Figure 4C demonstrates the results, showing low relative standard deviations (RSD) of 2.26% and 2.37% for inter-assays and intra-assays, respectively. These findings indicate that the developed biosensor exhibits high reliability and credibility in its performance.

### 3.6. Analysis of Pb^2+^ in Real Samples

The feasibility of the proposed biosensor was evaluated by detecting Pb^2+^ in real samples, including river water, drinking water, and tap water. Pb^2+^ was spiked into these samples at 0, 0.5, 50, and 250 nM concentrations. All samples were subjected to the same testing conditions, ensuring uniformity and comparability of the results, and the recoveries were determined and are presented in Table 3. The obtained recovery values ranged from 99.7 to 102.8%, indicating that the developed biosensor exhibited accurate and reliable detection of Pb^2+^ in complex sample matrices. The above results validate the practical applicability of the biosensor for environmental monitoring and water quality analysis.

## 4. Conclusions

We have developed a highly sensitive and specific colorimetric biosensor for the detection of Pb^2+^. The biosensor is based on a unique combination of GR5 DNAzyme, DNA walker, RCA, and G4/hemin DNAzyme. The mechanism involves the interaction between the target Pb^2+^ and the GR5 DNAzyme, which triggers the DNA walker to release cDNA from the SS. The liberated cDNA then initiates the RCA reaction, resulting in exponential amplification of the signal. The amplificated product, containing G-rich DNA sequences, binds to hemin to form G4/hemin, which exhibits peroxidase-like activity and catalyzes the oxidation of H_2_O_2_ and the substrate, generating a colorimetric signal. The developed biosensor offers several key advantages. Firstly, the utilization of the DNAzyme and DNA walker ensures high selectivity towards the target Pb^2+^ ions, reducing interference from other metal ions. Additionally, the RCA reaction and the inclusion of hemin further enhance the signal amplification, enabling high sensitivity with a detection limit of 3.3 pM. The biosensor exhibits a wide dynamic range of detection spanning from 10 pM to 1000 nM. Furthermore, the flexibility of DNA allows for the adaptation of the colorimetric biosensor to detect other targets by simply modifying the DNA sequence. This versatility enhances the potential applications of the colorimetric biosensor beyond Pb^2+^ detection.

## Figures and Tables

**Table 1:** The DNA sequences and modifications used in the study.

Probe Name	Sequence
SS	5′-biotin-TTT TTA CTG CTA CTC ACT AT/rA/GGA AGA GAT GAG ACT GAT-3′
FAM-SS	5′-biotin-TTT TTA CTG CTA CTC ACT AT/rA/GGA AGA GAT GAG ACT GAT-FAM-3′
ES	5′-CTC ATC TCT GAA GTA GCG CCG CCG TAT AGT GAG TAG CAG T-3′
cDNA	5′-GGA AGA GAT GAG ACT GAT-3′
TEMP	5′-P-ATC TCT TCC TTT TCC CAA CCC GCC CAA CCC TTT TTT TTT TCC CAA CCC GCC CAA CCC TTT TAT CAG TCT C-3′

**Table 2:** Comparison of the proposed assay with previous colorimetric biosensors for Pb^2+^ detection.

Recognition Element	Strategy	Linear Range	LOD	Ref.
GR5 DNAzyme	Functionalized AuNPs/magnetic nano-DNAzyme	0.1 nM–100 μM	32 pM	[46]
GR5 DNAzyme	Cleavage-induced HCR/AuNPs	0–1 nM	59.39 pM	[47]
GR5 DNAzyme	Cleavage-induced G4 formation/HCR/Duplex-hemin/TMB-H_2_O_2_	2.58–18 nM	0.77 nM	[48]
GR5 DNAzyme	Magnetic nano-DNAzyme walker/cleavage-induced RCA/G4/TMB-H_2_O_2_	0.01–1000 nM	3.3 pM	This work

**Table 3:** Recovery and RSDs of Pb^2+^ in real samples (*n* = 3).

Sample	Added Pb^2+^ (nM)	ICP-MS		This Work		
		Found Pb^2+^ (nM) *^a^*	RSD (%) *^b^*	Found Pb^2+^ (nM) *^a^*	RSD (%) *^b^*	Recovery (%)
River water	0	2.017	1.190	3.676	3.117	/
	0.5	2.648	0.873	4.188	2.362	102.416
	50	52.492	1.181	54.947	2.197	102.542
	250	252.187	0.945	249.084	2.112	98.163
Drinking water	0	/	/	0.205	4.331	/
	0.5	/	/	0.719	3.227	102.8
	50	50.284	0.523	51.034	2.233	101.658
	250	249.467	0.642	248.432	2.143	99.291
Tap water	0	/	/	0.346	3.627	/
	0.5	/	/	0.853	2.956	101.412
	50	49.953	0.452	49.833	2.423	98.974
	250	251.628	0.639	253.433	2.027	101.235

*^a^* Average of three determinations, *^b^* RSD: Relative signal deviation.

## Data Availability

Data will be made available on request.

## Supplementary Materials

The following supporting information can be downloaded at https://www.mdpi.com/article/10.3390/bios13080761/s1, Figure S1: Fluorescence assays of IMBs-DNA walker with different concentrations of Pb^2+^ (0, 1, 5, 10, 50, 100, 500, 1000 nM).
