# Supplementary material for: A Self-Assembled G-Quadruplex/Hemin DNAzyme-Driven DNA Walker Strategy for Sensitive and Rapid Detection of Lead Ions Based on Rolling Circle Amplification
Source: Biosensors (Basel). 2023 Jul 26;13(8):761. doi: 10.3390/bios13080761 (PMC10452914; doi:10.3390/bios13080761)
Supplement: Supplementary file 1 [file biosensors-13-00761-s001.zip › biosensors-2469360-supplementary.pdf]

biosensors

MDPI

Supporting Information

A Self-Assembled G-Quadruplex/Hemin DNAzyme-Driven DNA Walker Strategy for Sensitive and Rapid Detection of Lead Ions Based on Rolling Circle Amplification

Yuhan Wang [1], Jiaxuan Xiao [1], Xiaona Lin [2,3], Amira Waheed [2,3], Ayyanu Ravikumar [1], Zhen Zhang [1], Yanmin Zou [4,*] and Chengshui Chen [2,3,*]

1 School of Emergency Management, School of the Environment and Safety Engineering, Jiangsu University, Zhenjiang 212013, China
2 Department of Pulmonary and Critical Care Medicine, The Quzhou Affiliated Hospital of Wenzhou Medical University, Quzhou People's Hospital, Quzhou 324000, China
3 Key Laboratory of Interventional Pulmonology of Zhejiang Province, Department of Pulmonary and Critical Care Medicine, The First Affiliated Hospital of Wenzhou Medical University, Wenzhou 325000, China
4 School of Pharmacy, Jiangsu University, Zhenjiang 212013, China
* Correspondence: zouyanmin@ujs.edu.cn (Y.Z.); chenchengshui@wmu.edu.cn (C.C.); Fax: +86-577-55578186 (C.C.)

biosensors

MDPI

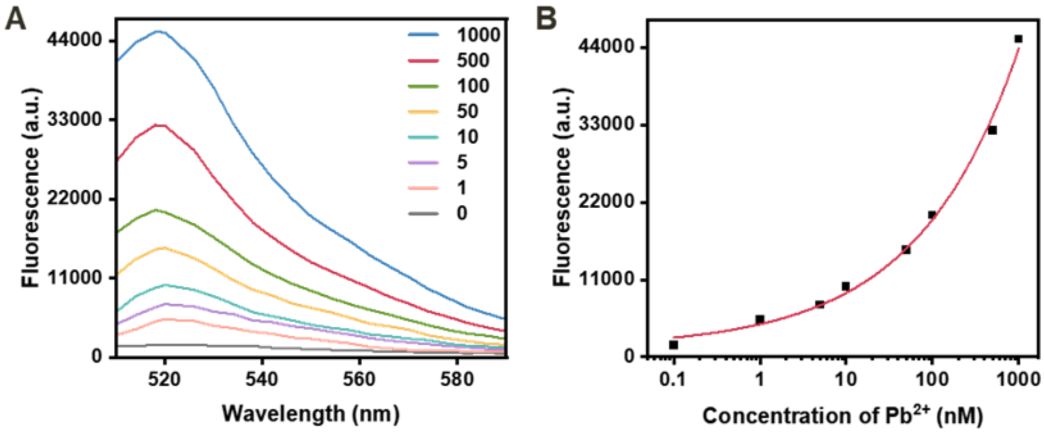

Figure S1. Fluorescence assays of IMBs-DNA walker with different concentrations of Pb^{2+} (0, 1, 5, 10, 50, 100, 500, 1000 nM).
